# Homonymous Hemianopsia Due to the Infarction in the Splenium of the Corpus Callosum

**DOI:** 10.7759/cureus.19574

**Published:** 2021-11-14

**Authors:** Masahito Katsuki, Hideaki Kato, Hiroshi Niizuma, Yoichi Nakagawa, Masahiro Tsunoda

**Affiliations:** 1 Neurosurgery, Senseki Hospital, Higashi-Matsushima, JPN; 2 Neurosurgery, Itoigawa General Hospital, Itoigawa, JPN; 3 Ophthalmology, Nakagawa Eye Clinic, Ishinomaki, JPN; 4 Ophthalmology, Tsunoda Ganka, Wakuya-cho, JPN

**Keywords:** atherosclerosis, visual information processing, hemianopsia, cerebral infarction, splenium

## Abstract

The precise functions of the splenium of the corpus callosum (CC) remain unclear, and infarction of this location manifests varied clinical symptoms. We describe a rare case of right homonymous hemianopsia resulting from pure infarction in the right-side splenium of the CC. An 85-year-old man presented with right homonymous hemianopsia lasting for a week. Diffusion-weighted imaging showed a high-intensity area in the right-side splenium of the CC and did not show any other lesions in other portions of the visual pathways. Magnetic resonance angiography demonstrated anterior and posterior cerebral arteries, indicating that no large vessel occlusion existed. The visual field examination revealed right homonymous hemianopsia. The diagnosis was atherothrombotic infarction in the splenium of the CC, which resulted in right homonymous hemianopsia. Two months later, T2-weighted imaging showed a high-intensity lesion localizing the right-side splenium with shrinkage of the lesion compared to that on the acute phase, and his visual field was slightly improved. There are few reports on the splenial infarction of the CC, and this is the first case manifesting as homonymous hemianopsia, to our knowledge. Our case might help to understand complicated visual information processing involving the splenium of the CC.

## Introduction

The corpus callosum (CC) is the biggest commissural fibrous bundle of the central nervous system and divided into four parts: the rostrum, genu, body, and splenium. The splenium is the thickest part of the CC, and the majority of the fibers extend posteriorly into the occipital lobes [1]. Infarctions of the CC are rare because of their abundant collateral blood supply from the anterior and posterior circulation through pericallosal anastomotic plexus [2]. The most common location of the CC infarction is the splenium [2]. However, clinical symptoms of the splenial lesion are variable, such as cognitive impairment, aphasia, and alien hand syndrome, and precise functions of the splenium remain unclear [3,4]. We herein describe a rare case of right homonymous hemianopsia due to splenial infarction of the CC. To our knowledge, this is the first case with right homonymous hemianopsia resulting from the splenial infarction of the CC.

## Case presentation

An 85-year-old right-handed man was referred for right homonymous hemianopsia lasting for a week by his ophthalmologist treating his glaucoma. He did not have any lifestyle-related diseases and take any medicines. The laboratory tests on admission revealed serum triglyceride level 133 mg/dL, high-density lipoprotein cholesterol level 32 mg/dL, low-density lipoprotein cholesterol level 59 mg/dL, glucose level 188 mg/dL, hemoglobin A1c level 6.4%, and other items were all within normal limits. He had habitual smoking and drinking. The Holter electrocardiogram did not find atrial fibrillation.

**Figure 1:**
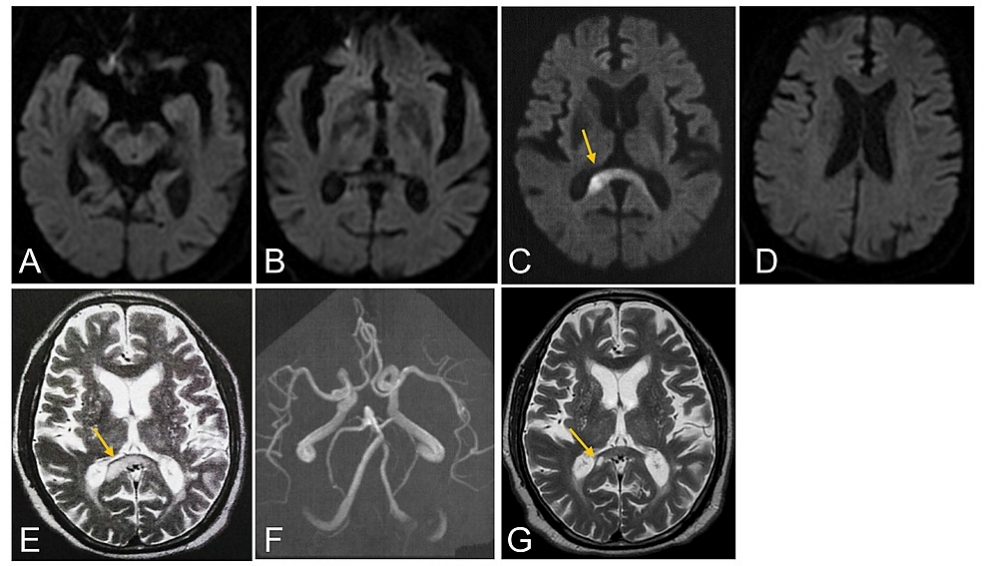
Magnetic resonance imaging Diffusion-weighted imaging on admission showed a high-intensity lesion spreading from the right-side splenium of the corpus callosum to the contralateral side (arrow in C), but did not show any other lesions in other portions of the visual pathways (A, B, D). T2-weighted imaging on admission showed the same finding (arrow in E). The magnetic resonance angiography revealed mild atherosclerosis (F). Two months later, T2-weighted imaging showed a high-intensity lesion, which became localized to the right-side splenium of the CC (arrow in G).

Diffusion-weighted imaging showed a high-intensity lesion mainly in the right-side splenium of the CC but the lesion extended to the contralateral side. No lesions were found in other portions of the visual pathways (Figure 1A-1D). The lesion was also evident on the T2-weighted imaging (T2WI) (Figure 1E). The magnetic resonance angiography revealed mild atherosclerosis (Figure 1F). The diagnosis was atherothrombotic infarction in the splenium of the CC, which resulted in right homonymous hemianopsia, and cilostazol was prescribed.

**Figure 2:**
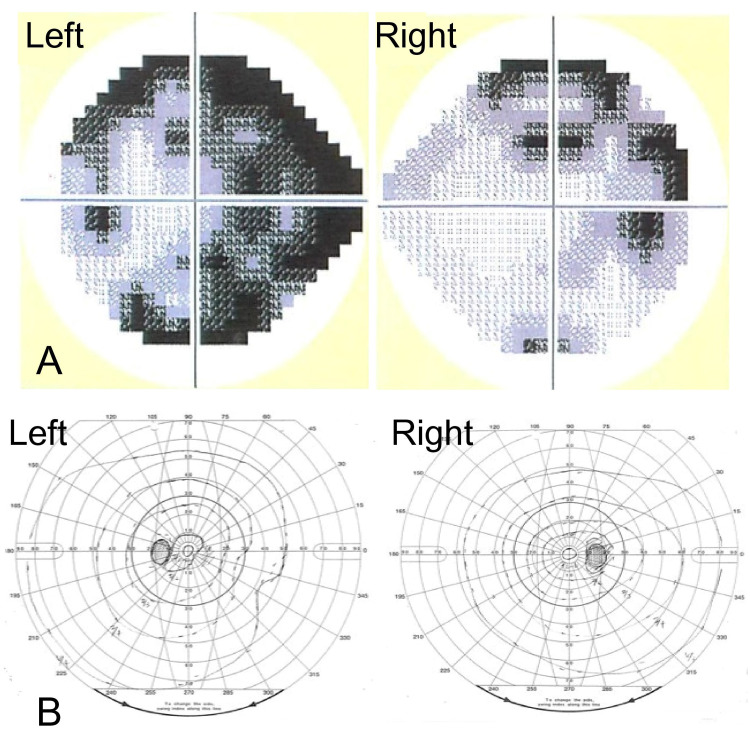
Visual field examination The visual field examination by an ophthalmologist on day 13 confirmed right homonymous hemianopsia with visual field narrowing due to glaucoma (A). The visual field examination 2 months later showed an improvement of his right homonymous hemianopsia (B).

The visual field examination on day 13 confirmed irregular pattern of visual field narrowing and defects, which is considered as a combination of right homonymous hemianopsia due to splenial infarction and visual field narrowing due to glaucoma (Figure 2A). No other ophthalmologic diseases were found. Two months later, the high-intensity lesion on T2WI significantly shrank and became localized to the small area of the right-side splenium of the CC (Figure 1G). The visual field examination confirmed an improvement of his homonymous hemianopsia (Figure 2B).

## Discussion

The visual pathway refers to the series of cells and synapses, which transmit visual signals from the environment to the brain for processing. This pathway begins with light striking the specialized nerve cells of the retina. It converts photons of light into electrochemical signals. The neural signals are transferred through the retinal layers to the optic nerve, optic chiasm, optic tract, lateral geniculate body, optic radiation, and visual cortex in the brain’s occipital lobe [5]. Each optic tract classically carries the contralateral half of the visual field, so the lesions on the optic tract, lateral geniculate body, optic radiation, and visual cortex result in homonymous hemianopsia. The primary cause of the homonymous hemianopsia is stroke (70%), and around 80% of its responsible lesions locate in the occipital lobes or the optic radiations [6]. However, our case had the right homonymous hemianopsia resulting from the splenial infarction with the intact optic tract, optic radiations, and occipital lobes.

The most common location of CC infarction is the splenium, but pure splenial infarction is rare. Because of the well-developed collateral circulation, CC infarction usually accompanies with additional lesion due to large vessel occlusion [2,7]. Therefore, there have been only 10 previous reports of pure splenium infarction [3,8-16] without any other lesions in the cortex nor optic tract (Table 1). No cases had homonymous hemianopsia, but four cases had metamorphopsia. Saito et al. hypothesized that the visual information would be processed in the bilateral temporal, parietal, and occipital lobes across the hemispheres and that metamorphopsia was caused by a disrupted transfer of visual information at the splenium and the major forceps [10]. Similarly, we hypothesized that right homonymous hemianopsia was caused by an interrupted transfer of visual information at the splenium of the CC across the hemispheres [17]. In other words, visibility would not be defined only in the unilateral striate cortex, but also in the bilateral cortexes across the hemispheres. However, this theory cannot explain the laterality. Further studies, including tractography and functional imaging, are expected.

**Table 1:** Previous reports on pure infarction in the splenium of corpus callosum ACA: anterior cerebral artery; ATBI: atherothrombotic cerebral infarction

Author	Year	Age	Sex	Laterality	Neurological symptoms	Etiology	Prognosis
Hashiguchi et al. [8]	2009	63	M	L	Loss of motivation, dysarthria, memory disturbance	ATBI	Not improved in 14 days
Katsura et al. [9]	2010	67	F	L	Right-sided metamorphopsia	ATBI	Not improved in 20 days
Saito et al. [10]	2014	61	F	R	Left-sided metamorphopsia	ATBI	Not improved in 2.5 years
Nagaishi et al. [11]	2015	78	F	R	Left-sided metamorphopsia	ATBI	Improved in 18 days
Chang and Huang [12]	2015	57	?	R	Hemiparesis, dysarthria	ATBI	Unknown
		43	?	L	Dizziness, memory disturbance	ATBI	Unknown
Li et al. [3]	2015	78	M	L	Hemiparesis, dysarthria	Undetermined	Unknown
		74	F	L	Hemiparesis, ataxia, hypesthesia	ATBI	Unknown
		61	F	Bilateral	Headache, vertigo, disturbance of consciousness	ATBI	Coma
Lai and Katirji [15]	2017	21	F	Center	Photophobia	Cerebral venous thrombosis	Improved in few days
Zhu et al. [13]	2018	53	M	R	Left-hand weakness	ACA dissection	Unknown
Barghouthi and El Husseini [16]	2018	67	F	L	Prosopometamorphopsia	Undetermined	Improved in a year
Zhang et al. [14]	2019	53	M	R	Unknown	ACA dissection	Unknown
		57	F	R	Dizziness	ATBI	Unknown
		Four others (not described)					
Our Case	2019	85	M	R	Right homonymous hemianopsia	ATBI	Slightly improved in 14 days

## Conclusions

We described a case with right homonymous hemianopsia resulting from the splenial infarction of the CC. We hypothesized that right homonymous hemianopsia was caused by an interrupted transfer of visual information at the splenium of the CC across the hemispheres. In other words, we hypothesized that the visibility would not be defined only in the unilateral striate cortex, but also in the bilateral cortexes across the hemispheres. Our case might help to understand complicated visual information processing involving the splenium of the CC. Further studies, including tractography and functional imaging, are expected.
